# Modeling climate change impacts on overwintering bald eagles

**DOI:** 10.1002/ece3.204

**Published:** 2012-03

**Authors:** Chris J Harvey, Pamela E Moriarty, Eric P Salathé Jr

**Affiliations:** 1 Northwest Fisheries Science CenterNOAA Fisheries, 2725 Montlake Blvd. E, Seattle, Washington 98112; 2 Biology and Mathematics DepartmentsKenyon College, Gambier, Ohio 43022; 3 Science & Technology Program, University of Washington-BothellBothell, Washington 98011-8246; 4 Climate Impacts Group, University of WashingtonP.O. Box 355672, Seattle, Washington 98195-5672

**Keywords:** Bald eagles, bioenergetics models, climate change, ecosystems, food webs, predation, regional climate models, salmon, scavenging

## Abstract

Bald eagles (*Haliaeetus leucocephalus*) are recovering from severe population declines, and are exerting pressure on food resources in some areas. Thousands of bald eagles overwinter near Puget Sound, primarily to feed on chum salmon (*Oncorhynchus keta*) carcasses. We used modeling techniques to examine how anticipated climate changes will affect energetic demands of overwintering bald eagles. We applied a regional downscaling method to two global climate change models to obtain hourly temperature, precipitation, wind, and longwave radiation estimates at the mouths of three Puget Sound tributaries (the Skagit, Hamma Hamma, and Nisqually rivers) in two decades, the 1970s and the 2050s. Climate data were used to drive bald eagle bioenergetics models from December to February for each river, year, and decade. Bald eagle bioenergetics were insensitive to climate change: despite warmer winters in the 2050s, particularly near the Nisqually River, bald eagle food requirements declined only slightly (<1%). However, the warming climate caused salmon carcasses to decompose more rapidly, resulting in 11% to 14% less annual carcass biomass available to eagles in the 2050s. That estimate is likely conservative, as it does not account for decreased availability of carcasses due to anticipated increases in winter stream flow. Future climate-driven declines in winter food availability, coupled with a growing bald eagle population, may force eagles to seek alternate prey in the Puget Sound area or in more remote ecosystems.

## Introduction

Bald eagle (*Haliaeetus leucocephalus*) populations, once decimated by stressors such as hunting, prey declines, predator control, habitat loss, and chemicals such as DDT, have largely recovered in the continental United States (USFWS 2007). This recovery follows a broad range of protective actions, and led to bald eagles being removed from the U.S. Endangered Species List in 2007. Though this recovery is clearly a conservation success story, growing numbers of bald eagles present challenges to natural resource management because bald eagles are large, mobile, endothermic animals with high metabolic demands (Stalmaster and Gessaman 1984) and diverse, opportunistic dietary habits (e.g., Stinson et al. 2007; Anthony et al. 2008). Bald eagles are top predators capable of depleting populations of seabirds (Parrish et al. 2001; Hayward et al. 2010), terrestrial mammals (Newsome et al. 2010, 2011; but see Hudgens et al. 2011), and possibly other prey resources.

**Figure 1:**
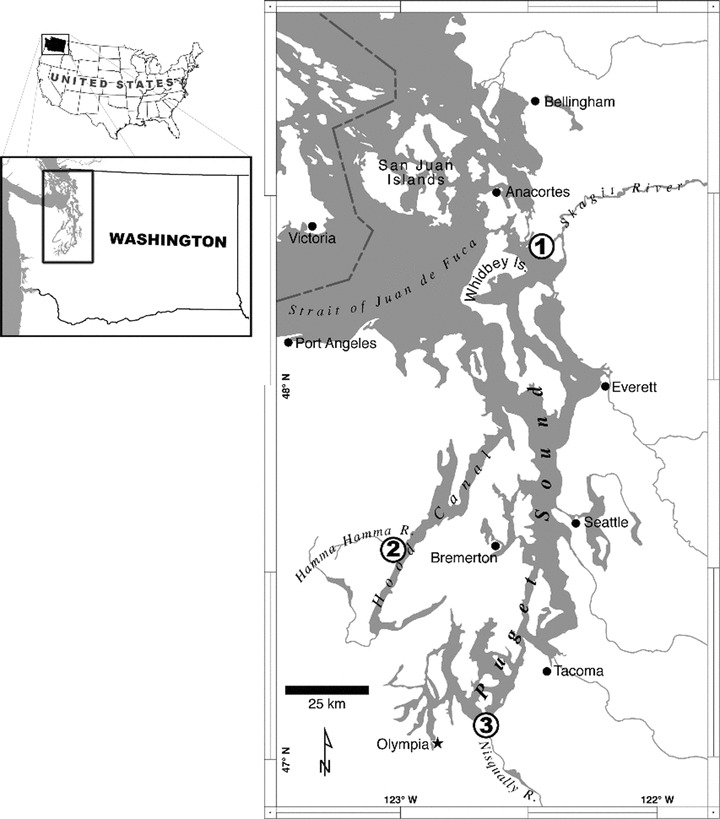
Map of Puget Sound. Study sites are: (1) Skagit River, (2) Hamma Hamma River, and (3) Nisqually River.

In Washington State, resident bald eagle numbers have increased dramatically. Stinson et al. (2007) reported a roughly 700% increase in the nesting population from 1981 to 2005, a population growth rate of 9% annually. Washington's bald eagle population grows two- to threefold during the winter months when birds from Canada, Alaska, and elsewhere in the western continental United States migrate to habitats in Washington (Stinson et al. 2007). In the Puget Sound region (Fig. 1), postspawned salmon carcasses, particularly chum salmon (*Oncorhynchus keta*), are major food sources during the overwintering period (Servheen 1975; Hunt et al. 1992; Dunwiddie and Kuntz 2001). Bald eagle numbers in Washington are expected to increase further in the coming decade (Stinson et al. 2007), placing additional demands on food resources.

Climate change in the Puget Sound region may alter energy requirements and food availability for overwintering eagles. For example, long-term climate change may affect air temperatures, wind velocity, cloud cover, and precipitation (Salathé et al. 2010), all of which influence bald eagle energy demands (Stalmaster and Gessaman 1984). Changes in river temperatures and flows may affect the abundance and accessibility of salmon carcasses (Glock et al. 1980; Stalmaster and Gessaman 1984; Hunt et al. 1992), which overwintering eagles feed upon. Such interactions may vary spatially, because different watersheds will likely have different numbers of eagles, salmon carcasses, and alternate prey. Also, regional climate change models predict substantial site-to-site variability in future air temperatures, precipitation, cloud cover, and wind speeds due to local factors such as topography, snow cover, and land–water contrasts (Salathé et al. 2010). Because bald eagles are mobile and opportunistic predators, poor overwinter feeding conditions in one area may lead them to seek alternate prey or move to other areas where feeding conditions are more favorable.

Here, we model the potential influence of climate change on feeding rates of overwintering bald eagles in three geographically distinct river basins of the Puget Sound region. For each basin, we compare estimates of eagle feeding under climate conditions from the past (1970s) and the projected future (2050s). We also estimate the rates at which salmon carcasses in each watershed break down under temperatures from the 1970s and 2050s, to determine the extent to which long-term climate variability will affect bald eagle food availability. We hypothesized that higher temperatures in the 2050s would lower the metabolic demands of overwintering eagles; that higher temperatures would also cause more rapid decomposition of salmon carcasses, reducing food availability; and that changes would differ by watershed due to local climate differences. Our approach, while theoretical, is intended to inform research and monitoring of bald eagle foraging ecology and behavior in relation to climate conditions, salmon abundance, and other prey populations in both nearby and remote ecosystems used by eagles.

## Methods

### Climate simulations

We used two regional climate simulations described by Salathé et al. (2010) to generate estimates for past (1970s) and future (2050s) states of climate conditions near the mouths of three rivers emptying into Puget Sound (Fig. 1). The simulations use the Weather Research and Forecasting (WRF) model, developed by the National Center for Atmospheric Research (NCAR). The WRF model in turn was forced by two global climate models: the NCAR Community Climate System Model, version 3 (CCSM3); and the Max Planck Institute, Hamburg, global climate model (ECHAM5/MPI-OM) (Roeckner et al. 1999, 2003; Marsland et al. 2003). Based on comparisons with a set of 19 global models, Mote and Salathé (2010) showed that both CCSM3 and ECHAM5 provide realistic simulations of the 20th century climate. Compared to the multimodel average for the Pacific Northwest, ECHAM5 projects a low temperature increase and a high precipitation increase while CCSM3 projects a relatively warmer and drier future. The WRF model is a state-of-the-art mesoscale numerical weather prediction system designed to serve both operational forecasting and atmospheric research needs (http://www.wrf-model.org). This model has been developed and used extensively in recent years for regional climate simulation (Leung et al. 2006). WRF is a nonhydrostatic model with multiple choices for physical parameterizations suitable for applications across scales ranging from meters to thousands of kilometers. The physics package includes microphysics, convective parameterization, planetary boundary layer, land surface models, and longwave and shortwave radiation. Details on model implementation for the present study are available in Salathé et al. (2010).

Both WRF simulations were run for 100 years (year 1 = 1970), and climate outputs were generated at 36-km grids (ECHAM5-WRF) or 20-km grids (CCSM3-WRF). Each model generated hourly estimates of the following variables of interest: air temperature at 2-m altitude, wind speed at 10-m altitude, total hourly precipitation, and downward longwave radiative flux. We compiled outputs for the months of December, January, and February, the months during which overwintering bald eagles are most abundant in the Puget Sound area (Stalmaster and Gessaman 1984; Stinson et al. 2007), for the decade of the 1970s and the decade of the 2050s. The outputs we compiled were specific to grid cells that centered nearest to the mouths of three Puget Sound tributaries: the Skagit, Hamma Hamma and Nisqually rivers (Fig. 1). These rivers were chosen because they are located in distinct subbasins of Puget Sound, and thus experience different local climate conditions; and because each supports large runs of late fall or winter chum salmon (spawning between late November and early March^1^).

### Bioenergetics modeling

Bald eagle winter feeding requirements were estimated using a bald eagle bioenergetics model developed by Stalmaster and Gessaman (1984). The model is a thermodynamic budget of energy gains (by consumption) and losses (due to respiration, waste production, and heat loss), which change as functions of body mass, activity level, and environmental variables such as temperature, precipitation, wind, and longwave radiation (related to cloud cover). All model functions, parameters, and parameter derivations are described in Stalmaster (1981) and Stalmaster and Gessaman (1984); for this paper, the function of interest is the core equation for daily food consumption (*C*, g·bird^–1^·day^–1^):


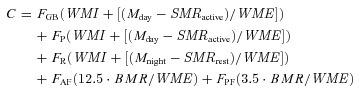
1
where *F* is the fraction of daily time spent feeding or waiting on gravel bars (*F*_GB_), daytime perching (*F*_P_), nighttime roosting (*F*_R_), in active flight (*F*_AF_), or in passive flight (*F*_PF_); *WMI*= wet matter intake (g·kg^–1^·day^–1^); *M*= metabolic heat production (kJ·kg^–1^·day^–1^) during daytime (*M*_day_) or nighttime (*M*_night_) hours; *SMR*= standard metabolic rate (kJ·kg^–1^·day^–1^) when active (*SMR*_active_) or resting (*SMR*_rest_); *WME*= diet energy density (kJ·kg^–1^); *BMR*= basal metabolic rate (kJ·kg^–1^·day^–1^), and 12.5 and 3.5 are metabolic rate multipliers for flapping and gliding flight, respectively. The terms *M*_day_, *M*_night_, *SMR*_active_, and *SMR*_rest_ represent functions influenced by temperature, and *M*_day_ and *M*_night_ are also influenced by wind speed, precipitation rate, and net longwave radiative exchange between the eagle and its environment (Stalmaster and Gessaman 1984).

We used the model to estimate daily food consumption from December to February for each year of each time period. In the manner of Stalmaster and Gessaman (1984), our model simulates consumption rates of a 4.5-kg adult eagle, feeding on chum salmon carcasses (*WME*= 3.764 kJ·kg^–1^) and consuming enough each day to maintain constant mass. Simulations were run at daily time steps and driven by inputs of daily average air temperature, daily average wind velocity, total daily precipitation, and daily average longwave radiation. In total, 120 simulations were run (two regional climate models, three rivers, two decades, 10 years per decade). Because the Stalmaster and Gessaman (1984) model discretely models diurnal and nocturnal eagle metabolism and activity levels, we weighted the daily average temperatures, winds, and longwave radiation by the total hours of light and dark each day.

As noted above, our models follow the assumption that chum salmon carcasses are the only food consumed during the winter months. This is certainly an oversimplification. Although salmon carcasses are likely the major winter food source for bald eagles in the Puget Sound area, due to their nutritional value and relative ease of procurement, eagles will emigrate or switch from scavenging to predation when salmon carcasses become limiting (Hunt et al. 1992; Stinson et al. 2007). Our focus on carcass consumption allows us to compare conditions in different decades and river basins to determine when and where such limitation is most likely; this, in turn, would indicate when bald eagles are most likely to affect other species (through switching from scavenging to predation) or systems (through relocation to better foraging habitats).

### Carcass decomposition rates

Stalmaster and Gessaman (1984) described temporal decomposition of chum salmon carcasses that were either submerged or out of the water. Carcasses declined in both mass and nutritional quality through time, and temperature appeared to positively influence the rate of decomposition, although they did not present enough temperature and decomposition rate data to define a relationship. We examined several published studies of salmonid carcass decomposition in or near stream habitats (Table 1), and from each study we compiled or derived estimates of the daily rate of decomposition, –*k*:

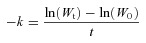
2
where *W* is salmon dry weight at time 0 or time *t*, and *t* is elapsed time in days. We then estimated the statistical relationship between –*k* and temperatures from the studies. Because the studies differed considerably in terms of initial carcass size, temperature, location, and the assemblage of scavengers present, the relationship between –*k* and temperature should only be regarded as representative of the process of carcass decomposition and not necessarily accurate for chum salmon carcasses in Puget Sound.

**Table 1:** Temperature-specific rates of decomposition (*k*) of carcasses of chum salmon (*Oncorhynchus keta*), rainbow trout (*O. mykiss*), and pink salmon (*O. gorbuscha*). Values of *k* were derived from equation (2). Carcass decomposition occurred either in air or under water, with minimal exclusion of scavenging invertebrates.

Species	Temperature (°C)	*k*	Exposure	Source
Chum salmon	−0.5	−0.0018	Air	Stalmaster & Gessaman 1984
Rainbow trout	4.2	−0.0155	Water	Minshall et al. 1991
Chum salmon	4.3	−0.0314	Air	Stalmaster & Gessaman 1984
Rainbow trout	4.4	−0.0210	Water	Fenoglio et al. 2010
Chum salmon	8.0	−0.0236	Water	Stalmaster & Gessaman 1984
Chum salmon	8.0	−0.0358	Water	Stalmaster & Gessaman 1984
Rainbow trout	8.6	−0.0479	Water	Minshall et al. 1991
Pink salmon	9.5	−0.0330	Water	Chaloner et al. 2002
Rainbow trout	16.0	−0.0570	Water	Fenoglio et al. 2005

We next estimated biomass of a hypothetical run of chum salmon over the December–February period. All salmon runs were comprised of ∼41,000 individuals; each weighed 3.652 kg, of which 16% was assumed to be dry matter at the time of death (Stalmaster and Gessaman 1984). The first salmon entered the stream on December 1 and the run proceeded for 10 weeks; entry was normally distributed. The run size and phenology was patterned after a carefully monitored chum salmon population, the one that returns to Kennedy Creek, a Puget Sound tributary near the Nisqually basin (Washington Department of Fish and Wildlife, unpubl. data^2^). We assumed a life span of 10 days on the spawning grounds (Salo 1991), after which salmon died and entered the carcass pool. Decomposition of individuals was calculated daily, using the relationship between temperature and –*k*, based on the daily average air temperatures for each river, year, and climate model. That is, we specifically estimated decomposition of carcasses pulled out of the water by eagles for consumption. Total dry biomass of carcasses was summed each day until February 28, after which eagles were assumed to leave the area, consistent with the bioenergetics model exercises.

### Statistics

To determine if any differences in climate variables or bald eagle consumption rates were statistically significant, we used analysis of variance (ANOVA). For climate model outputs, we first averaged data from each winter simulation on a monthly basis so that we might more clearly distinguish long-term signals (climate) from highly correlated daily variability (weather). Monthly mean precipitation values were square root transformed to meet assumptions of normality (Kolmogorov–Smirnov test, *n*= 360, *P*-value = 0.417). We used ANOVA to test the hypothesis that climate variables varied by decade, month, climate model (all fixed effects), and site (random effect), and examined pairwise comparisons with Bonferroni post-hoc tests (used due to small sample sizes of 10 winters per decade). For bald eagle bioenergetics estimates, we pooled model outputs by month and calculated the average daily kilojoules of energy consumed in each simulation. We again used ANOVA to test the hypothesis that consumption varied by decade, month, climate model (fixed effects), and site (random effect), and used Bonferroni tests for post-hoc pairwise comparisons.

## Results

### Climate change projections

**Figure 2:**
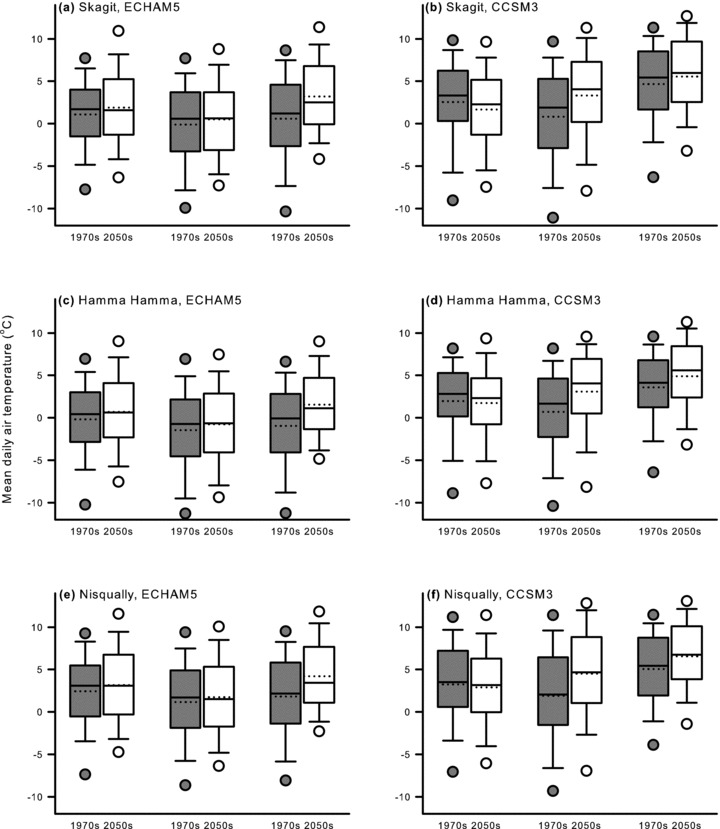
Mean daily air temperatures by site, model, and decade. Plots indicate median (central solid line), grand mean (central dashed line), 25th and 75th percentiles (boxes), 10th and 90th percentiles (whiskers), and 5th and 95th percentiles (solid circles).

According to regional climate models, air temperatures at all sites were projected to increase between the 1970s and the 2050s (Fig. 2 and Table 2). That was particularly true in January and February, as indicated by interaction effects in the ANOVA (decade × month interactions; both Bonferroni *P*-values < 0.001). The extremes (5th and 95th percentiles) were also warmer in the 2050s than the 1970s in nearly all cases (Fig. 2). Air temperatures for the Nisqually River generally were the warmest and for Hamma Hamma were the coldest (Fig. 2; ANOVA, Bonferroni *P*-values < 0.001). Also, site-specific temperature outputs from the CCSM3 model were significantly warmer than the ECHAM5 model, often by several degrees and particularly in January and February (Fig. 2 and Table 2; Bonferroni *P*-values < 0.001). This indicates that the ECHAM5 model has a greater “cold bias” than the CCSM3 model, despite the fact that both models tracked observed regional temperatures (1970–1999) quite well (see details in Salathé et al. 2010, their Fig. 1).

**Table 2:** Analysis of variance (ANOVA) results for comparisons of mean monthly air temperature and square root-transformed precipitation from Puget Sound regional climate model outputs. Effects were decade (1970s vs. 2050s), month (December, January, February), climate model (ECHAM5 vs. CCSM3; see text for details), site (Skagit, Hamma Hamma, Nisqually), and interactions. Statistically significant factors are in bold. The *r*^2^ values indicate the total variation in the monthly means explained by the model.

	Temperature; *r*^2^= 0.336				Precipitation; *r*^2^= 0.277			
Source	df	Sum of squares	*F*-ratio	*P*-value	df	Sum of squares	*F*-ratio	*P*-value
Decade	**1**	**160.4**	**938.4**	**0.001**	1	0.005	0.500	0.553
Month	**2**	**306.8**	**175.5**	**<0.001**	**2**	**0.084**	**8.724**	**0.035**
Model	**1**	**386.0**	**31.815**	**0.030**	1	0.372	0.439	0.576
Site	**2**	**239.8**	**14.693**	**<0.001**	**2**	**0.617**	**12.611**	**<0.001**
Decade × month	**2**	**60.935**	**141.1**	**<0.001**	**2**	**0.049**	**43.693**	**0.002**
Decade × model	1	0.156	0.491	0.556	1	0.014	5.777	0.138
Decade × site	2	0.342	0.021	0.979	2	0.020	0.414	0.662
Month × model	**2**	**90.502**	**628.5**	**<0.001**	**2**	**0.108**	**10.521**	**0.026**
Month × site	4	3.497	0.107	0.980	4	0.019	0.196	0.940
Model × site	2	24.268	1.487	0.228	**2**	**1.693**	**34.594**	**<0.001**
Decade × month × model	**2**	**64.015**	**128.5**	**<0.001**	2	0.020	4.291	0.101
Decade × month × site	4	0.864	0.026	0.999	4	0.002	0.023	0.999
Decade × model × site	2	0.636	0.039	0.962	2	0.005	0.099	0.906
Month × model × site	4	0.289	0.009	1.000	4	0.021	0.210	0.933
Decade × month × model × site	4	0.997	0.031	0.998	4	0.009	0.094	0.984
Error	324	2643.5			324	7.929		

**Figure 3:**
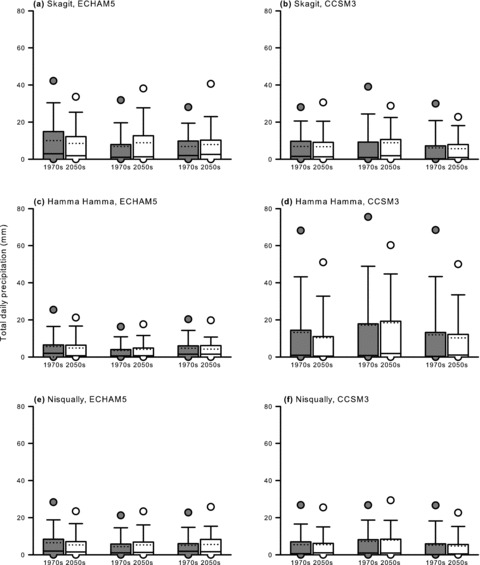
Mean daily precipitation by site, model, month, and decade. Plots indicate median (central solid line), grand mean (central dashed line), 25th and 75th percentiles (boxes), 10th and 90th percentiles (whiskers), and 5th and 95th percentiles (solid circles).

Predicted changes in precipitation from the 1970s to the 2050s were inconsistent (Fig. 3). That is, precipitation did not uniformly increase, decrease, or remain relatively stable across sites, months, or models. There was no statistical difference in mean monthly precipitation from the 1970s to the 2050s (ANOVA, Table 2), and a mildly significant difference by month (no pairwise differences; Bonferroni *P*-values > 0.2). Precipitation did vary by site (significantly lower in the Nisqually; Bonferroni *P*-values ≤ 0.001) and model (significantly lower in ECHAM5). The most notable difference was in the outputs of the two climate models for the Hamma Hamma site, where the ECHAM5 model predicted lower precipitation rates and variability than did the CCSM3 model (Fig. 3c–d; Bonferroni *P*-value < 0.001). It is worth noting that these estimates are intended to represent winter precipitation falling in the spatial grid cells near the three river mouths, but are not necessarily indicative of winter precipitation at the scale of the three rivers’ entire watersheds; that is, the projected river levels and discharge rates may not be directly related to the precipitation estimates shown in Figure 3.

For the sake of brevity, we report here only that downward longwave radiative fluxes are projected to increase mildly at all sites in the 2050s relative to the 1970s, particularly during February (data not shown). This implies slightly less winter cloud cover in the 2050s. Wind speeds showed no significant changes from the 1970s to the 2050s, regardless of month, climate model, or site (data not shown, again for brevity).

### Bald eagle bioenergetics

**Figure 4:**
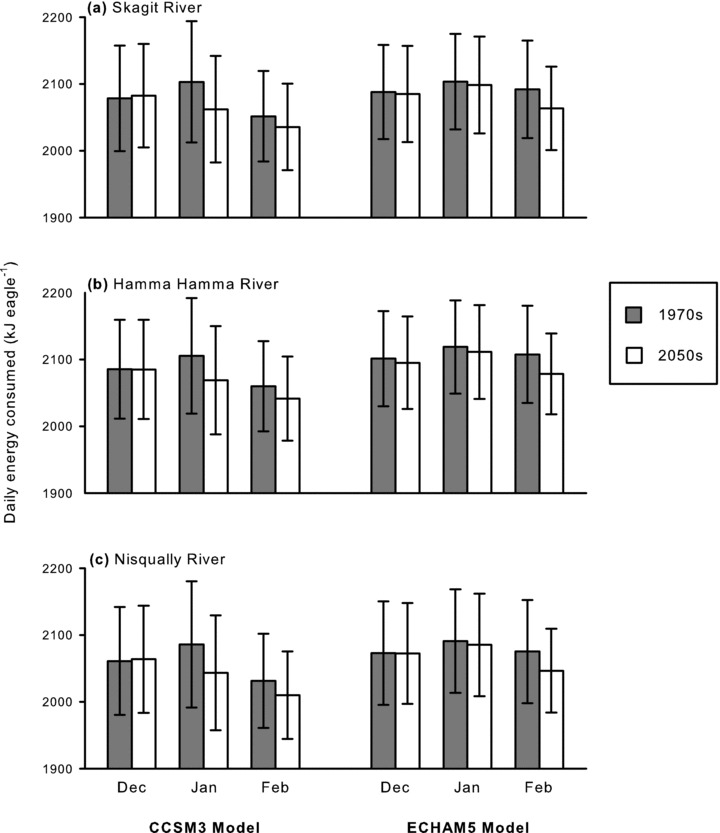
Mean daily bald eagle energy consumption (± standard deviation), calculated with bioenergetics models and plotted by site, model, month, and decade.

Bald eagles that overwinter in the three catchments will experience little change in energy requirements as a result of climate change, according to bioenergetics models. As expected, the warmer, drier conditions of the 2050s lowered daily energy requirements relative to the 1970s (ANOVA, Table 3), but the decreases were small, regardless of catchment or climate model (Fig. 4). When daily energy requirements were summed for the full winter, the average decline in total energy requirement from the 1970s to the 2050s was <1%. Daily energy requirements were slightly lower in February than other months (Fig. 4 and Table 3; Bonferroni *P*-value < 0.001). There were several significant interactions between month and other effects (Table 3); most notably, daily consumption during the month of January declined in the 2050s relative to the 1970s, and also declined during February in the 2050s relative to February in the 1970s (Bonferroni *P*-values < 0.01). Energy requirements were lower in CCSM3 simulations compared to ECHAM5 simulations, and energy requirements differed by site, due to slightly lower consumption in Nisqually simulations than in Hamma Hamma or Skagit models (Fig. 4 and Table 3; Bonferroni *P*-values ≤ 0.001).

**Table 3:** ANOVA results for comparisons of daily energy consumption by overwintering bald eagles in Puget Sound, as derived from bioenergetics model outputs. Main effects were as in Table 2. Statistically significant factors are in bold. The model explained 33.3% of total variation in the data (i.e., *r*^2^= 0.333).

Source	df	Sum of Squares	*F*-ratio	*P*-value
Decade	**1**	**22,325.7**	**1266.0**	**0.001**
Month	**2**	**65,090.6**	**212.6**	**<0.001**
Model	**1**	**51,641.8**	**100.4**	**0.010**
Site	**2**	**43,650.8**	**16.233**	**<0.001**
Decade × month	**2**	**10,419.3**	**145.5**	**<0.001**
Decade × model	**1**	**857.4**	**18.641**	**0.050**
Decade × site	2	35.271	0.013	0.987
Month × model	**2**	**12,858.8**	**1128.2**	**<0.001**
Month × site	4	612.2	0.114	0.978
Model × site	2	1029.2	0.383	0.682
Decade × month × model	**2**	**8789.4**	**271.8**	**<0.001**
Decade × month × site	4	143.2	0.027	0.999
Decade × model × site	2	91.988	0.034	0.966
Month × model × site	4	22.796	0.004	1.000
Decade × month × model × site	4	64.668	0.012	1.000
Error	324	435,610.6		

Although the climate-driven differences between decades were statistically significant, the very small decrease in energy requirements appears to have little actual ecological significance: according to energy-to-mass conversion factors in Stalmaster and Gessaman (1984), a 1% decrease in total consumption amounts to roughly 0.1 to 0.2 fewer salmon carcasses over the course of a winter in the 2050s compared to the 1970s.

### Carcass decomposition

The literature review (Table 1) generated a positive relationship between temperature and salmonid carcass decomposition rates. A linear regression estimating –*k* as a function of temperature (°C) produced a strong fit (slope =–0.0032, intercept =–0.0072, *r*^2^= 0.80, *P* < 0.001, *n*= 9). We applied daily climate model temperature estimates from each river to this relationship in order to estimate and compare salmon decomposition.

**Figure 5:**
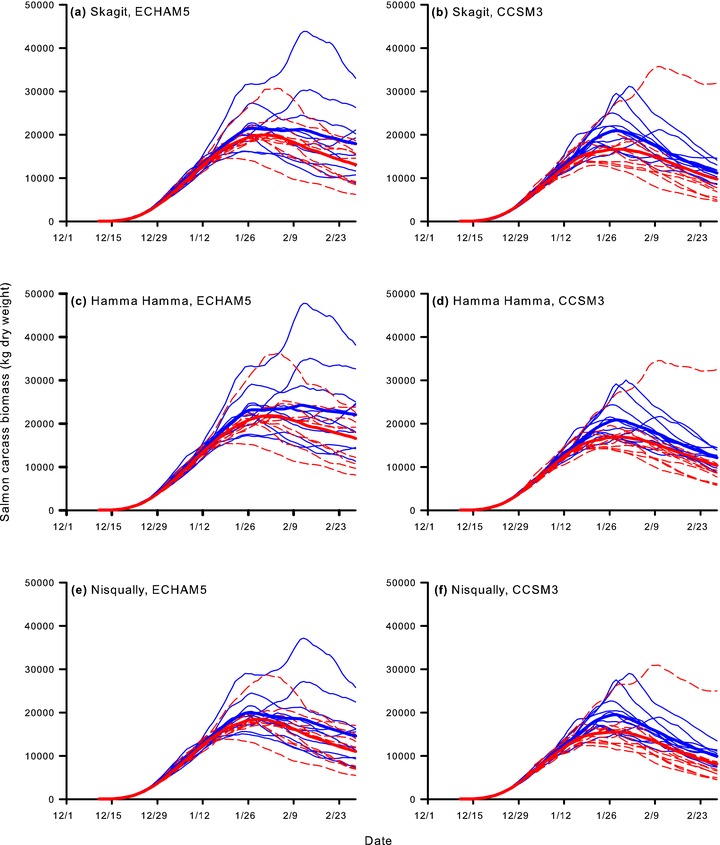
Estimated daily chum salmon carcass biomass (dry weight), plotted by site and model. Each line represents total carcass biomass from a hypothetical run of 40,560 adult chum salmon during the 1970s (blue lines) or 2050s (red lines). Biomass estimates were based on new additions of fresh carcasses and temperature-mediated daily decomposition of carcasses. Heavy central lines represent mean values by decade.

Across all catchments and models, the average rate of decomposition was generally greater in the 2050s than in the 1970s, resulting in lower availability of carcasses in the 2050s (Fig. 5). The rate of chum salmon carcass decomposition was a function of year, the underlying climate model, and to a lesser extent catchment; on average, total carcass biomass for the December–February period decreased 12–13% in the Skagit and Hamma Hamma rivers and 11–14% in the Nisqually River, depending on which regional climate model was used. Interannual variability increased from mid-January through the end of February as the incoming supply of fresh carcasses declined and temperature-driven decomposition became the main factor driving total carcass biomass. In general, the ECHAM5 model resulted in greater variability in decomposition rates and carcass biomass trends than the CCSM3 model (Fig. 5).

Interdecadal differences within individual river catchments were most apparent when the CCSM3 climate model was used (Fig. 5b, d, and f). Based on temperatures from that model, total carcass biomass trends from the 1970s had relatively little overlap with trends from the 2050s, and differences increased later in the winter. For example, during the month of February, mean 2050s carcass biomass ranged from 12% to 19% lower than mean 1970s biomass, depending on Julian day and river (Fig. 5b, d, and f, heavy lines), but this comparison was influenced heavily by a single extreme cold year from the 2050s that resulted in anomalously high carcass biomass. When that anomalous year was removed, mean 2050s carcass biomass was 23–37% lower than mean 1970s biomass, depending on Julian day and river. When the ECHAM5 model was used, carcass biomass was again greater in the 1970s, but there was somewhat greater interdecadal overlap (Fig. 5a, c, and e). For example, during February, mean 2050s carcass biomass was 5–27% lower than mean 1970s biomass, depending on day and river (Fig. 5a, c, and e, heavy lines), but again there was an anomalously cold year, this time from the 1970s, that influenced the average difference. When that year was omitted, the mean difference shrank to 0–19%, depending on the day and river.

Carcass availability varied by river to some extent; the main difference was that for the standardized chum salmon run, the Nisqually River typically ended with the lowest available carcass biomass, regardless of decade and especially when the CCSM3 model was used (Fig. 5 and Table 4). The Hamma Hamma River had slightly more carcass biomass than the Skagit when the ECHAM5 model was used, regardless of decade, but when the CCSM3 model was used, the Hamma Hamma and Skagit rivers had very similar biomasses.

**Table 4:** Estimated mean final carcass biomass (on February 28), in kg dry weight, for hypothetical chum salmon runs under the 12 combinations of climate conditions (by decade, model, and site). Parenthetical values are proportions relative to the Hamma Hamma River value within each model/decade combination.

Model/site	1970s		2050s	
ECHAM5				
Skagit	17,884	(0.81)	13,069	(0.79)
Hamma Hamma	22,074	(1.00)	16,592	(1.00)
Nisqually	14,548	(0.66)	11,016	(0.66)
CCSM3				
Skagit	11,170	(0.92)	9771	(0.94)
Hamma Hamma	12,101	(1.00)	10,352	(1.00)
Nisqually	9881	(0.82)	8089	(0.78)

## Discussion

According to our analysis, the effects of climate change on overwintering bald eagle bioenergetics in the Puget Sound region will be outweighed by the effects on bald eagle food supply. Warmer winters in the 2050s caused a slight decrease in eagle metabolism, but sharply accelerated decomposition of salmon carcasses, their main food source. We further expect reduced food quality; Stalmaster and Gessaman (1984) observed a steady temporal decline in energy content (kJ·g^–1^) of chum salmon carcasses, and the microbial and invertebrate activity that reduces energy content should increase at higher temperatures (DeVault et al. 2004). Although precipitation at our sites was projected to decrease slightly, winter flows in most Puget Sound rivers are expected to increase due to higher temperatures, land use changes, and reduced water storage in mountain snowpack (Elsner et al. 2010; Cuo et al. 2011). Higher flows may reduce carcass availability (Glock et al. 1980; Hunt et al. 1992) and visibility (Patterson et al. 2007), and have been correlated with reduced bald eagle foraging success in other systems (Brown et al. 1998). Moreover, bald eagle densities in Washington are expected to continue increasing (Stinson et al. 2007), which may lower individual feeding efficiency (Stalmaster and Gessaman 1984).

Because eagle metabolism was insensitive to changes in climate variables, energy requirements differed only slightly among the three basins. However, carcass decomposition rates showed much greater spatial variability. Overwintering bald eagles congregate around rivers with abundant salmon carcasses, and frequently relocate to take advantage of stream-to-stream differences in salmon abundance and run timing (Hunt et al. 1992; Watson and Pierce 2001). Thus, regional carrying capacity for overwintering bald eagles likely depends on chum salmon population sizes, run timings, carcass availability (related to river flow), and carcass decomposition rates in each of the major chum salmon rivers. Maintaining diversity in run timing among chum salmon stocks may mitigate some of the risk of food limitation caused by high flows or faster decomposition rates.

How climate change will affect chum salmon populations is unclear. We are unaware of research on how climate affects chum salmon run timing. One study on Japanese chum salmon suggests that a warming climate will lead to larger run sizes of smaller bodied adults (Seo et al. 2011), while another predicts that climate-driven alterations in bioenergetics and migration routes will cause major declines in Japanese chum stocks (Kishi et al. 2010). In the northeast Pacific, Mueter et al. (2005) found a weak positive relationship between chum salmon survival and sea surface temperatures during their early marine phase. Chum population sizes in the northwestern United States and British Columbia are negatively correlated with spring precipitation (Fukuwaka et al. 2011); however, the ECHAM5 and CCSM3 model projections disagree on future trends for spring precipitation around Puget Sound (Salathé et al. 2010). Ruggerone and Goetz (2004) hypothesized that climate change will exacerbate competition among salmon, while Irvine and Fukuwaka (2011) concluded that salmon are nearing carrying capacity in the North Pacific due to basin-wide increases in pink (*O. gorbuscha*) and chum salmon. The ultimate effects of climate on chum salmon runs will likely be driven by a combination of global, regional, and population-specific factors (Fukuwaka et al. 2011).

In addition to unknown climate impacts on chum salmon, several other sources of uncertainty must be considered when interpreting our results. Although the bioenergetics model was quite insensitive to climate change, it assumes uniform diets (salmon carcasses) and bald eagle demographics (all birds = 4.5 kg). Both factors are considerably more variable than assumed and would affect population-level energy requirements. Also, the carcass decomposition rate estimate was drawn from a number of studies under different conditions. While it is reasonable to assume that a warmer climate will accelerate carcass decomposition, the actual rate will vary as a function of not only temperature but also carcass size, exposure to water and sunlight, microbial activity, and feeding by other scavengers (Chaloner et al. 2002; Fenoglio et al. 2010). Finally, climate change projections incorporate multiple sources of uncertainty, including the future emissions of greenhouse gases, the sensitivity of global climate models to greenhouse gas forcing, and the regional response to global climate change as represented by the downscaling method (Mote and Salathé 2010). For example, Mote and Salathé (2010) examined low-emission and high-emission projections from an ensemble of 21 global climate models, and used statistical downscaling methods to derive finer scale climate projections for the northwestern continental United States. Temperature changes are fairly consistent among models, with low-emissions scenarios producing delayed warming compared to high-emissions scenarios. Projected changes in annual precipitation, however, varied widely among emissions scenarios and across climate models, with uncertainties comparable to the range of natural variability. Moreover, the process of developing regional climate change models (Salathé et al. 2010), from which we derived the site-specific climate estimates used in this study, is much more computationally expensive than statistical downscaling. Thus, we have only considered two climate scenarios here, which cannot account for variability across models to the extent that a larger ensemble would allow. Nevertheless, the scenarios we have used in this study are illustrative of the likely sensitivity of bald eagle bioenergetics to climate change.

### Potential impacts on communities

Hunt et al. (1992) found that as salmon carcasses became less available in the Skagit River, overwintering eagles moved to shoreline areas along Puget Sound and the Strait of Georgia, where their prey was primarily birds and marine fishes. Using satellite tags, Watson and Pierce (2001) showed that overwintering eagles in the Skagit River area also spent parts of the winter east of the Cascade Mountains, north into parts of British Columbia and Alberta, and south into Oregon and California. Thus, a climate-related decline in salmon carcass availability could have measurable impacts on other systems if eagles relocate earlier or more often in search of winter forage. Relocation to new foraging sites or a transition to avian prey would involve an increase in active flying, which is a considerable energetic expense that can markedly increase total feeding requirements (Stalmaster and Gessaman 1984).

How climate change will affect the ecological role of large scavenger/predators is uncertain, due to the underlying complexity of the communities they inhabit (DeVault et al. 2003; Wilmers and Post 2006; Wilson and Wolkovich 2011). Understanding the relationship between bald eagles, salmon carcasses, and other food sources is important, given the central role salmon carcasses play in aquatic and terrestrial ecosystems proximate to spawning grounds (Gende et al. 2002), and the ability of eagles to disrupt prey populations such as seabirds (Parrish et al. 2001; White et al. 2006). If overwintering bald eagles respond to reduced carcass availability by switching prey and/or foraging habitat, then they will likely rely on a prey base that is broader (geographically, taxonomically, or both) than at present. This may result in greater predation on overwintering waterfowl, scavenging of upland carrion, and displacement of other scavengers and predators. Thus, regional climate-mediated dynamics between overwintering bald eagles and Puget Sound salmon populations may ultimately affect the functioning of other ecosystems that bald eagles exploit.

Our findings are grounded in predictive physiological and climate models, and are best viewed as hypotheses about likely relationships between bald eagles, climate, and winter food resources. Validation of our work will require monitoring or focused empirical study. Valuable insights could be gained through well-conceived study designs that simultaneously measure multiple variables (e.g., temperature, flow, salmon run sizes, carcass deposition, and decomposition rates) along with tracking of eagle behavior (residence time near rivers, tracking of relocation to alternate foraging areas, monitoring of prey selection), particularly in winters with contrasting weather conditions (e.g., El Niño vs. La Niña years). Such work would be especially effective if the ecology of other carcass decomposers and scavengers, ranging from microbes and invertebrates to terrestrial mammals, was also considered so that the scope of impact could be evaluated at a community scale.
